# Differential Nutrient Uptake by Saltmarsh Plants Is Modified by Increasing Salinity

**DOI:** 10.3389/fpls.2021.709453

**Published:** 2021-07-29

**Authors:** Raquel Carmona, Rocío Muñoz, F. Xavier Niell

**Affiliations:** Departamento de Ecología, Facultad de Ciencias, Universidad de Málaga, Málaga, Spain

**Keywords:** *Atriplex*, *Arthrocnemum*, competition, eutrophication, nutrient uptake, salinity, salt marsh, *Sarcocornia*

## Abstract

In Southern European estuaries and associated salt marshes, the anthropogenic nutrient inputs, together with longer drought periods, are leading to increasing eutrophication and salinization of these coastal ecosystems. In this study, uptake kinetics of ammonium, nitrate, and phosphate by three common plants in Palmones salt marsh (Southern Spain), *Sarcocornia perennis* ssp. *alpini, Atriplex portulacoides*, and *Arthrocnemum macrostachyum* were measured in hydroponic cultures. We also determined how these uptakes could be modified by increasing salinity, adding NaCl to the incubation medium (from 170 to 1,025 mM). Kinetic parameters are analyzed to understand the competition of the three species for nutrient resources under realistic most frequent concentrations in the salt marsh. These results may also be useful to predict the possible changes in the community composition and distribution if trends in environmental changes persist. *Atriplex portulacoides* showed the highest V_max_ for ammonium, the most abundant nutrient in the salt marsh, while the highest affinity for this nutrient was observed in *A. macrostachyum*. Maximum uptake rates for nitrate were much lower than for ammonium, without significant differences among species. The highest V_max_ value for phosphate was observed in *A. macrostachyum*, whereas *A. portulacoides* presented the highest affinity for this nutrient. High salinity drastically affected the physiological response of these species, decreasing nutrient uptake. *Sarcocornia perennis* ssp. *alpini* and *A. macrostachyum* were not affected by salinity up to 510 mM NaCl, whereas *A. portulacoides* notably decreased its uptake capacity at 427 mM and even withered at 1,025 mM NaCl. At current most frequent concentrations of ammonium and phosphate in the salt marsh, *S. perennis* ssp. *alpini* is the most favored species, from the nutritional point of view. However, *A. portulacoides* could enhance its presence if the increasing ammonium load continues, although a simultaneous salinization would negatively affect its nutritional physiology.

## Introduction

Salt marshes around the world are under threat, and understanding the responses to major environmental disturbances is critical to maintaining the health and conservation of these coastal ecosystems (Millennium Ecosystem Assessment, 2005; IPCC, 2014). These ecosystems are highly productive coastal wetlands that provide important ecosystem services, such as storm protection for coastal cities, nutrient removal, and carbon sequestration (Deegan et al., 2007, 2012). Salt marshes play a crucial role in nutrient cycles in transitional waters (Adam, 2002). In this sense, they have been described as important sources of nutrients, from their own produced and degraded organic matter, which is transformed into inorganic substances and exported to the estuarine waters (Nixon, 1980; Odum et al., 1995), as well as active sinks of coarse organic matter that is sequestered in the sediment by diagenesis (Nedwell, 2000; Turner et al., 2002). Over the last decades, coastal marshes of Southern Europe have been reduced by more than 60% (Lotze et al., 2006; Airoldi and Beck, 2007) due to multiple stress factors, mainly eutrophication and the sea-level rise (Zaldívar et al., 2008; Deegan et al., 2012). A concomitant salinization of coastal wetlands is occurring at an unprecedented rate and can be accelerated due to regional and global climate change (Herbert et al., 2015). This is the case of the salt marshes in Mediterranean climates, which are bearing more extreme events, as rising temperatures, floods, higher evaporation rates, and a decrease in rainfall and river flow (Ibañez et al., 1999; Álvarez-Rogel et al., 2000; Redondo-Gómez et al., 2007a; González-Alcaraz et al., 2014; Hassan et al., 2016; Cramer et al., 2018; Cañedo-Argüelles et al., 2019; Pereira et al., 2019; Vélez-Martín et al., 2020).

There is a wealth of information on how nutrient loading can affect growth and productivity of saltmarsh plants (Valiela et al., 1976; Morris et al., 2013; Wong et al., 2015; Johnson et al., 2016; Redelstein et al., 2018). There are also numerous studies describing how salinity influences their production and distribution (Redondo-Gómez et al., 2006, 2007b, 2010; Woo and Takekawa, 2012; Herbert et al., 2015; Ferronato et al., 2018; Vélez-Martín et al., 2020). Studies on the interactive effects of both factors, salinity and nutrients, on the physiological performance have focused on the genus *Spartina* (Mendelssohn and Morris, 2000; Alberti et al., 2010; Merino et al., 2010; Alldred et al., 2017; MacTavish and Cohen, 2017), showing different responses. For example, high salinity inhibits ammonium assimilation by *Spartina alterniflora* (Bradley and Morris, 1991); small additions of ammonium can offset salinity stress (MacTavish and Cohen, 2017), but a lack of a combined effect was also observed (Alldred et al., 2017). On the other hand, reports on nutrient uptake kinetics *per se* of halophytic species are scarce (Bradley and Morris, 1991; Mozdzer et al., 2010, 2011; Cott et al., 2018). As far as we know, there is no information on nitrogen and phosphorus uptake kinetics in the genera *Sarcocornia, Atriplex*, and *Arthrocnemum*, common in Mediterranean salt marshes, except for the work of Muñoz and Niell (2009).

The Palmones river estuary and associated salt marsh, where this study was carried out, is the last wetland of the eastern Atlantic before reaching the Mediterranean coasts, and it is a good example of the estuaries of the subarid areas of southern Spain. It is located in an industrial and densely populated area, where eutrophication has enhanced in the last 30 years, mainly due to a lower river discharge by the construction of a dam in its upper part and climatic changes, which led to severe and long drought seasons. Both situations have affected the flow of water in the river between the dam and the estuary, and input of nutrients with tidal flux has contributed to increase eutrophication (Clavero et al., 1997, 1999; Niell et al., 2005). In relation to nitrogen loading, nitrate is present at very low concentrations in the estuary and in the sediment interstitial water of the salt marsh, whereas high levels of ammonium have been measured (Palomo and Niell, 2009). Therefore, nitrogen is not limiting plant growth as in other salt marshes (Valiela et al., 1978; Mendelssohn, 1979; Pennings et al., 2005; Crain, 2007).

A progressive salinization has also been observed (Clavero et al., 1999; Rubio et al., 2003; Sánchez de Pedro et al., 2016), following the trend of other Mediterranean coastal wetlands, as mentioned above. This coastal ecosystem has been intensively studied, in relation to the impacts of environmental changes and human activities on nutrient cycles and ecophysiology of the different macrophytes inhabiting them (Pérez-Lloréns and Niell, 1990; Clavero et al., 1997, 1999, 2000; Hernández et al., 1997; Palomo et al., 2004; Niell et al., 2005; Palomo and Niell, 2009; Ruiz-Nieto et al., 2014; Sánchez de Pedro et al., 2016). In this salt marsh, the dominant plant species belong to the genera *Sarcocornia, Atriplex*, and *Arthrocnemum*, occurring in distinct zones of the salt marsh, following an elevation gradient (Palomo and Niell, 2009). It is well-known that plant zonation in salt marshes is mainly determined by salinity and tidal inundation gradient, as abiotic factors (Chapman, 1974; Colmer and Flowers, 2008; Flowers and Colmer, 2008) but also by biological interactions (Adams, 1963; Pennings and Callaway, 1992; Bertness and Ewanchuk, 2002).

The aim of this study was to determine nutrient uptake capacity in three dominant species of Palmones salt marsh and how it could be affected by salinity. For this, we obtained kinetic parameters of ammonium, nitrate, and phosphate and the uptake rates at most frequent nutrient concentrations in the salt marsh and under a wide range of increasing salinities. We further analyzed the results to explain the current distribution and possible changes in the plant community in response to global change factors, such as increasing eutrophication and salinization.

## Materials and Methods

### Site Description

**Figure 1:**
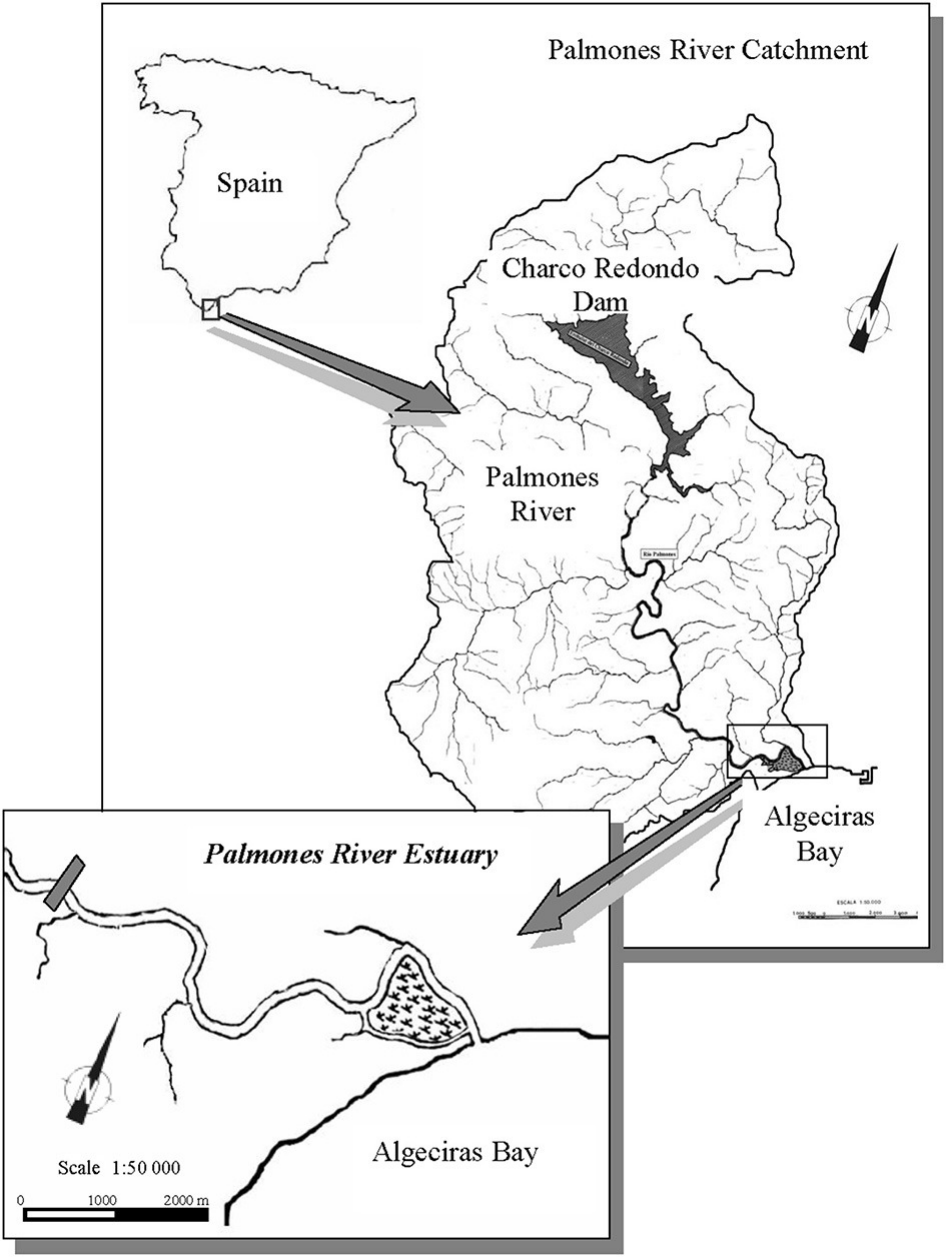
Map of the location of the study site.

The Palmones river estuary (36°10′17″N, 05°26′28″E) is located in the Algeciras Bay, South of Spain (Figure 1). The estuary, defined as partially mixed, has a surface area of 3.75 km^2^ and 1.2 m of tidal amplitude (Clavero et al., 1997, 1999). Salinity decreases from the mouth to the upper estuary and depends on seasonally variable freshwater discharges (Avilés and Niell, 2005). The salt marsh has an area of 1 km^2^, and the sediment accumulation average is 0.9 cm year^−1^ (Rubio et al., 2003). This salt marsh has been cataloged as SCI (site of community interest), SACs (special areas of conservation), and SPA (special protection areas for birds). The studied species are among the most abundant halophytes in the salt marsh: *Sarcocornia perennis* ssp. *alpini* (Lag.), Castroviejo, *Atriplex portulacoides* (L.) Aellen [syn. *Halimione portulacoides* Aellen] and *Arthrocnemum macrostachyum* (Moric.) C. Koch, which are positioned 35–65 cm above the lower spring minimal tide level (LSMTL). In the outer zone, closer to the estuary, the vegetation is dominated by *Sarcocornia perennis* ssp. *alpini*, which grows together with *Atriplex portulacoides*, while, in the drier inner salt marsh, the dominant species is *Arthrocnemum macrostachyum*. In the middle zone, *S. perennis* ssp. *alpini* and *A. macrostachyum* are found, whereas *A. portulacoides* is scarcely observed.

### Nutrient Concentration and Salinity of the Sediment

From October 2009 until July 2010, sediment samples were taken every 3 months to determine nutrient concentration at three zones along the seawater–land transect in the salt marsh: outer (36°10′207″N, 5°26′454″W), middle (36°10′20″N, 5°26′454″W), and inner (36°10′193″N, 5°26′451″W) zones, as suggested by Bouchard et al. (1998) and Bouchard and Lefeuvre (2000). The outer zone is the closest to the estuarine seawater and the inner, the furthest one (Figure 1). At each zone, samples were collected by means of cores of a 2.5-cm diameter inserted in the sediment (*n* = 4). Once in the laboratory, slices of 2 cm of sediment were separated down to 12 cm depth and centrifuged at 5,000 rpm to obtain the interstitial water, in which concentrations of ammonium, nitrate, and phosphate were measured after filtration through Whatman GF/C filters of 25 μm. Salinity was also measured *in situ* along the mentioned transect by means of a specific probe (CRISON CM 35, model 5060; Crison Instruments, Barcelona, Spain).

### Plant Collection and Acclimation Conditions

**Figure 2:**
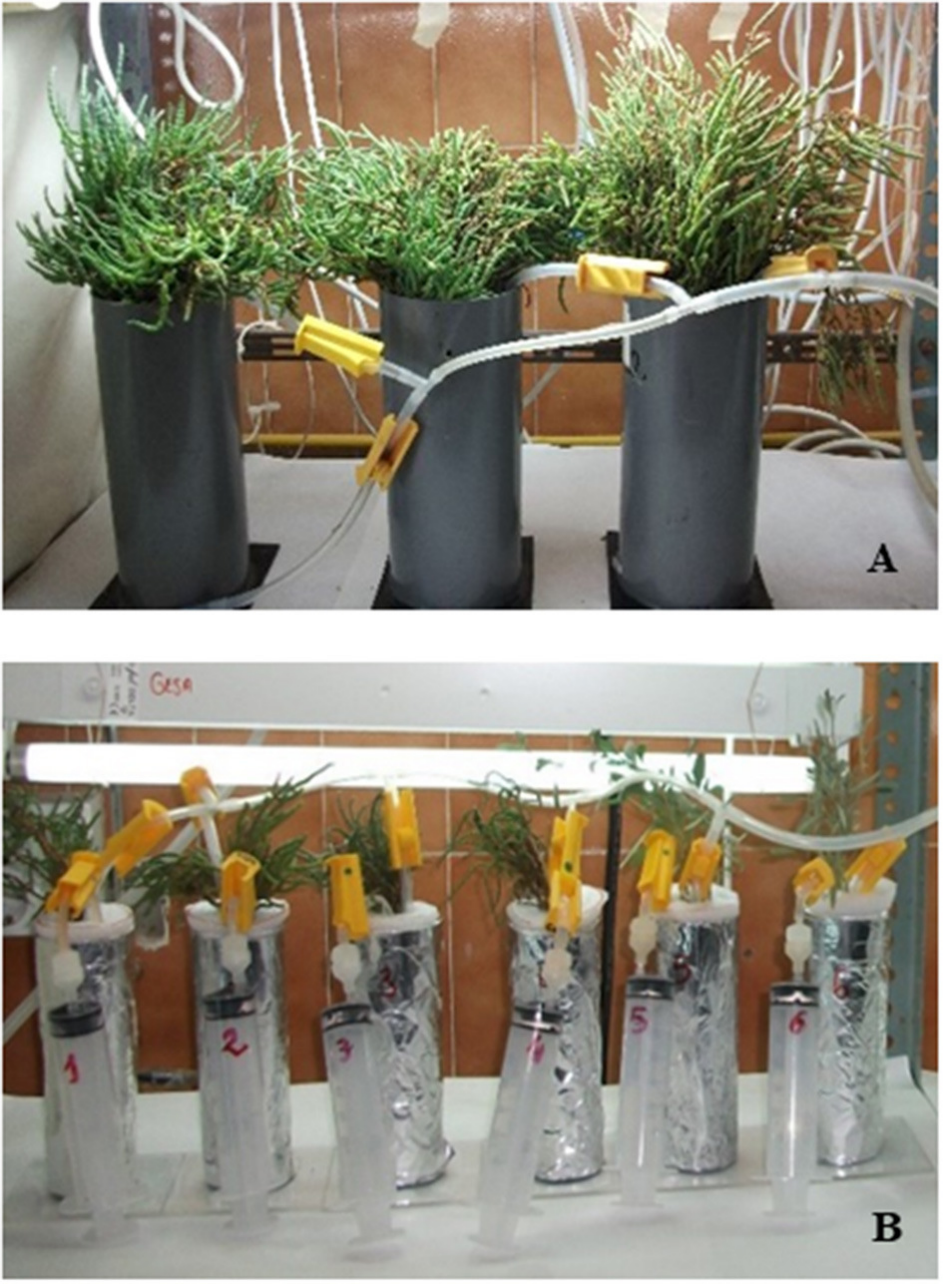
Photographs of the studied species grown in hydroponic cultures **(A)** and in the nutrient uptake experiments **(B)**.

Young healthy plants, smaller than 20 cm in height, were carefully removed from the salt marsh and transported with a portion of their own rhizosphere in a cooler to the laboratory. Then they were gently shaken and washed with a Hoagland modified medium (Epstein, 1972) until the roots appeared free of soil. This medium has been successfully used for acclimation and maintenance of chenopods by Palomo (2004) and Muñoz and Niell (2009). Cleaned plants were maintained in hydroponic cultures in cylindrical PVC containers of 16 cm high × 8 cm diameter, with 1 L of culture medium (Figure 2A). The plants were held in place by insertion into a tight-fitting stopper (granular polyurethane, 2-cm thick) that prevented exchange between the culture solution and the atmosphere. Before running the uptake experiments, plants were acclimated in a walk-in cold room chamber at 25°C and 200 μmoles photons m^−2^ s^−1^ of white light provided by fluorescent lamps (cool daylight, FL8T8/D Sylvania), with a photoperiod of 16:8-h light: darkness, cultured in Hoagland-modified medium (Epstein, 1972). Culture medium was changed weekly, and the mixing of nutrients was guaranteed by bubbling softly to avoid damage of roots. The pH was adjusted and maintained at 6.1, reproducing the values recorded in the salt marsh sediment. After 4 weeks, the plants produced roots, and they were considered to be ready for conducting the uptake experiments.

### Experimental Design

For the uptake experiments, plants were transferred to glass cylindrical containers with 220 ml of a basic medium, containing 500 mM NaCl, 10 mM KCl, 12 mM CaCl_2_, 55 mM MgCl_2_, 2 mM NaHCO_3_, and buffers 5 mM MES and 5 mM BIS-TRIS propane, to maintain a pH of 6.1 under the same temperature and irradiance as for acclimation. Preliminary control measurements with these containers proved that this material did not interfere with nutrient uptake by plants. Two probes were introduced into the containers, one for aeration and the other one to extract medium samples by means of a syringe (Figure 2B). Prior to uptake experiments, plants were starved for N or P for 5 days. The uptake kinetics were determined in four independent replicates for each nutrient, after the addition of 100, 400, 600, 800, 1,000, and 1,500 μM of NH_4_Cl, 2.5, 5, 10, 15, 20, and 50 μM of KNO_3_ and 5, 10, 20, 50, 100, and 150 μM of KH_2_PO_4_. The depletion of nutrients in the medium was determined taking samples after 15 min, 30 min, 1, 3, 5, and 24 h. These water samples were stored at −20°C until nutrient analyses.

Another set of experiments was conducted to test the effect of salinity on nutrient uptake rates. For this, plants were acclimated progressively, adding to the medium 0.1, 1, 20, 50, 100, 170, 250, 427, 510, and 1,025 mM NaCl to avoid osmotic shock. Finally, plants remained at the selected experimental salinities (170, 427, 510, and 1,025 mM NaCl) for 1 week before carrying out the uptake measurements, as previously described. These concentrations are equivalent to salinities of 10, 25, 30, and 60 psu. At the end of the incubation period, roots were separated from the plants and dried at 60°C until constant weight (48 h), and the uptake rates were expressed on the base of that dry weight.

### Nutrient Analyses and Uptake Rates

Water samples were analyzed in an automated nutrient analyzer QuAAtro AQ2 AACE (Seal Analytical Ltd, Fareham, UK), using the standard methods for ammonium (Slawyk and and MacIsaac, 1972), nitrate (Shinn, 1941; Wood et al., 1967), and phosphate (Fernández et al., 1985).

Uptake rates were calculated as the slope of the linear regression of the time-course depletion curve of each nutrient and expressed as μmol g^−1^ of root dry weight min^−1^. The relationship between the uptake rates and nutrient concentration was fit to the following Michaelis-Menten function, modified according to Barber (1979) and Brix et al. (1994, 2002), using the software KaleidaGraph 4.0 (Synergy Software):

(1) $$\begin{array}{r} \text{V}={\text{V}}_{\text{max}}\left[\text{X}-\text{CP}/\left({\text{K}}_{\text{m}}+\left(\text{X}-\text{CP}\right)\right)\right], \end{array}$$

where:

V is the uptake rate at a given (X) concentration;

X is the initial nutrient concentration in the medium;

V_max_ is the maximum uptake rate of the nutrient;

CP is the compensation point for the nutrient, which means that, at lower concentrations, there is no net nutrient uptake;

K_m_ is the nutrient half-saturation concentration or the concentration to reach half the V_max_.

### Statistical Analyses

Differences among the three species in the uptake kinetic parameters in the basic medium were tested by one-way ANOVAs. For the salinity experiment, differences among salinities for each species were also determined by one-way ANOVAs. In all cases, Tukey's HSD-test was used for *post hoc* comparisons. The significance level was set at α = 0.05. Statistical analyses were performed, using SigmaPlot 11.0 (Systat Software Inc., Chicago, IL, USA).

## Results

### Nutrient Concentrations in the Sediment

**Figure 3:**
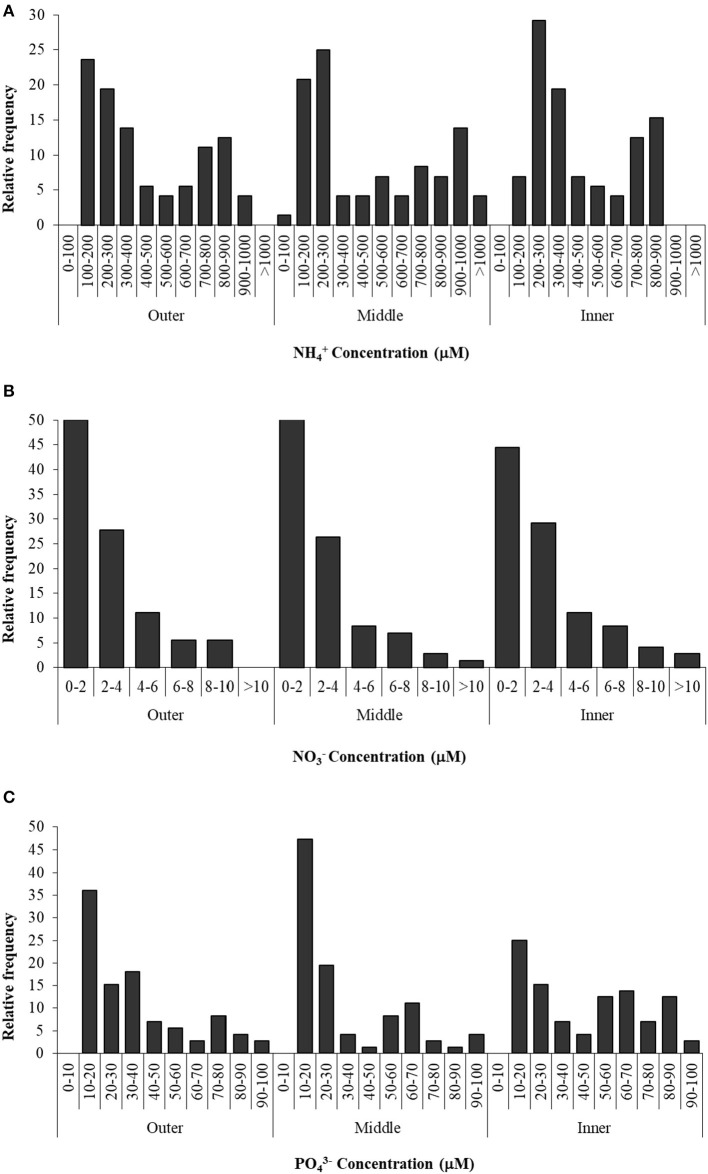
Ammonium **(A)**, nitrate **(B)**, and phosphate **(C)** concentration in three zones of Palmones salt marsh soil along the seawater-land gradient (outer, middle, and inner zones), expressed as the observed frequency of each concentration range.

Nutrient concentrations in the sediment interstitial water of the Palmones salt marsh fluctuated over the studied period (Figure 3). Values were expressed as the observed relative frequency (a probability percentage) of each concentration range, along the seawater–land gradient (outer, middle, and inner zones). Ammonium concentration ranged from 100 μM to 1.5 mM, showing a similar bimodal distribution in the three zones (Figure 3). The most frequent values were around 100–300 μM in the outer and middle zones of the salt marsh and 200–400 μM in the inner one, with a submode (around 25% of the samples) between 700 and 900 μM. Nitrate concentration varied from 0 to 13 μM, with around 50% of the samples within the lowest concentration range (0–2 μM), regardless of the zone, whereas concentrations higher than 4 μM were very infrequent (< 25% of probability). Soluble phosphate concentration varied between 10 and 100 μM, and the most frequent values were observed in the lowest range (10–20 μM) in the three zones (Figure 3). On the other hand, the probability to find higher concentrations (50–90 μM of phosphate) in the inner zone was two times that in the outer and middle ones.

### Salinity in the Sediment

**Figure 4:**
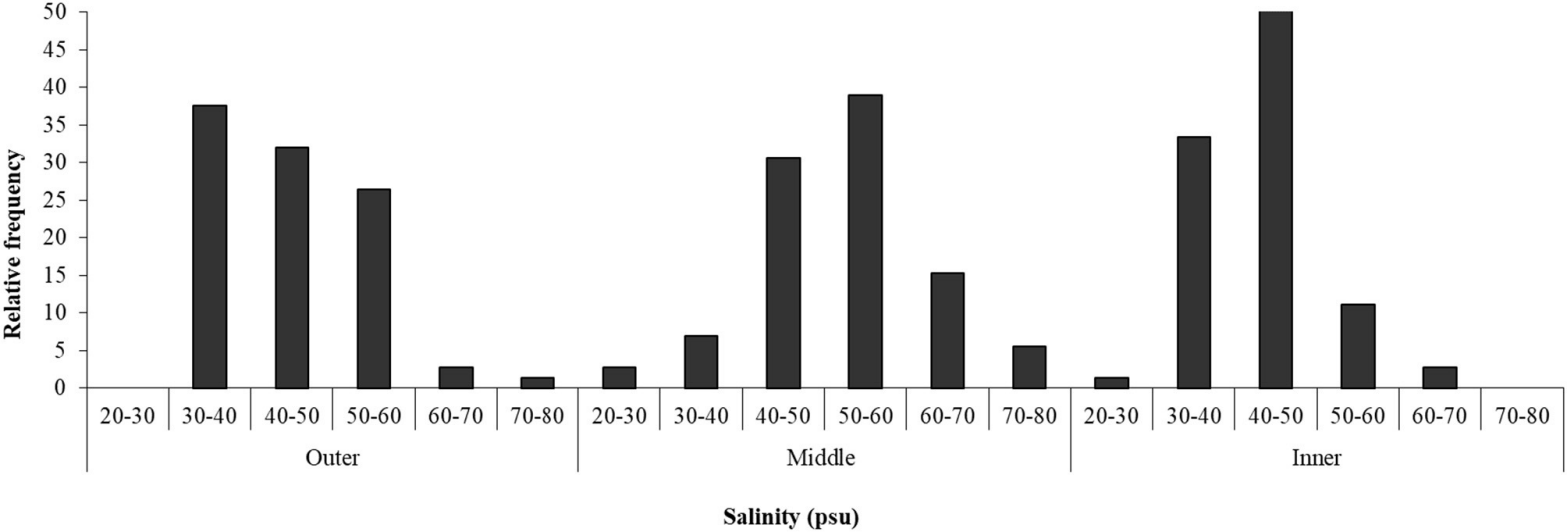
Salinity in three zones of Palmones saltmarsh soil along the seawater-land gradient (outer, middle, and inner zones), expressed as the observed frequency of each concentration range.

Salinity in the sediment interstitial water varied within the range between 20 and 80 psu (Figure 4). In the outer zone of the salt marsh, most samples (64%) showed a salinity in the classes of 30–40 and 40–50 psu, while, in the middle zone, 39% of the samples were in the class of 50–60 psu and 31% in that of 40–50 psu. In the inner part of the salt marsh, the most frequent salinity values (in 51% of the samples) were between 40 and 50 psu.

### Nutrient Uptake

**Figure 5:**
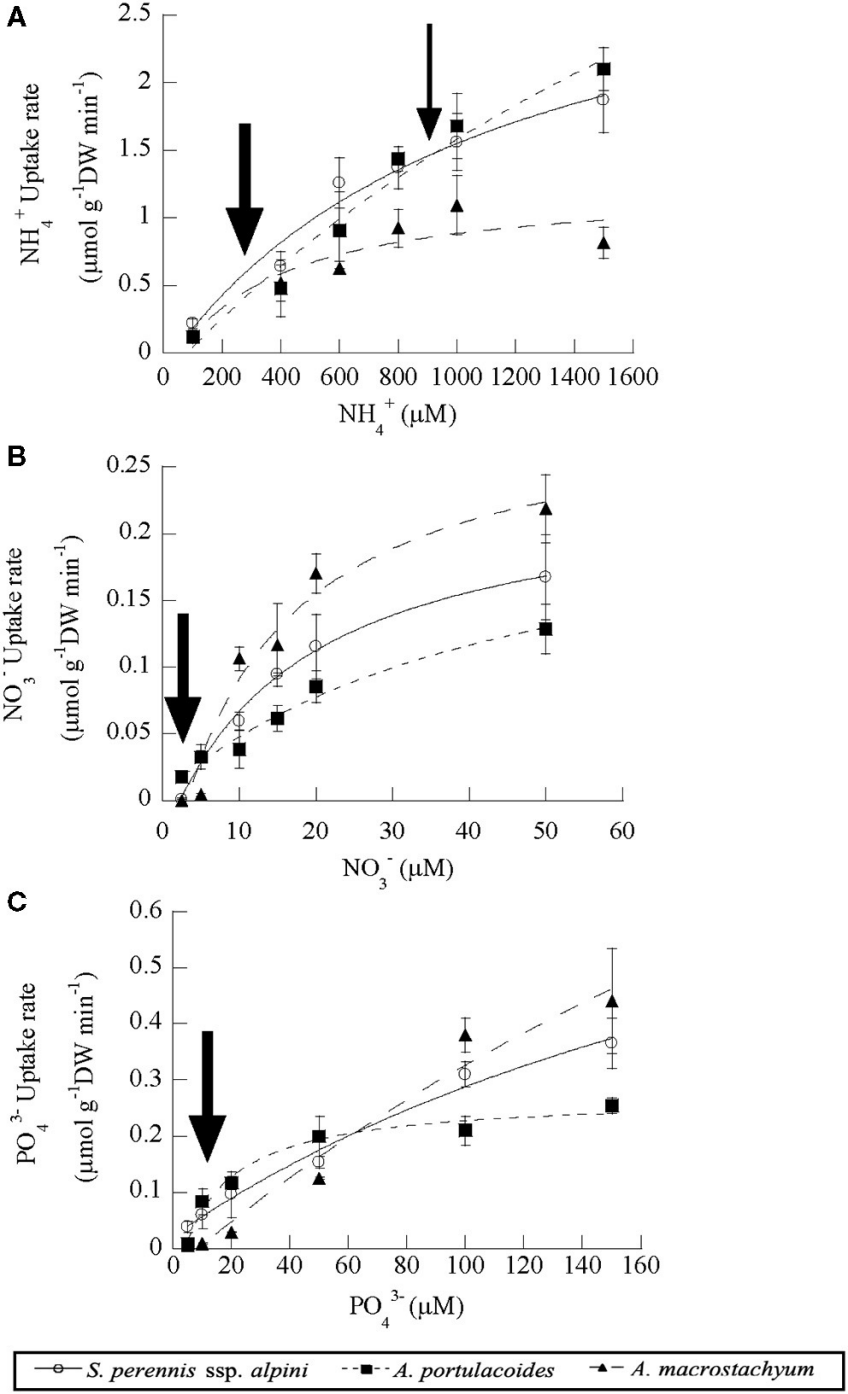
Uptake kinetics of ammonium **(A)**, nitrate **(B)** and phosphate **(C)** in the three species of the study (*S. perennis* ssp. *alpini, A. portulacoides* and *A. macrostachyum*). Bars represent standard deviation (*n* = 4). The thick and thin arrows point out the uptake at modal and submodal concentrations of nutrients, respectively.

Uptake kinetics saturated and fitted the Michaelis–Menten modified model in the three species of the study for all the nutrients assayed (Figure 5). *Atriplex portulacoides* showed the highest V_max_ for ammonium, being 2-fold greater than in *S. pernnis* ssp. *alpini* and almost 6-fold than in *A. macrostachyum* (Table 1). On the other hand, the highest affinity for this nutrient was observed in *A. macrostachyum*, while *S. perennis* ssp. *alpini* presented a CP, approximately half the ammonium concentration than the other two species. Uptake rates for nitrate were lower than for ammonium, especially in *A. portulacoides* (almost 30-fold lower), and there were no differences in V_max_ among species (Table 1). On the contrary, *A. portulacoides* had the lowest affinity and CP for nitrate. The highest V_max_ value for phosphate uptake was observed in *A. macrostachyum*, whereas *A. portulacoides* was the most efficient species, with K_m_ and CP values much lower than in *S. perennis* ssp. *alpini* and *A. macrostachyum* (Table 1).

**Table 1:** Kinetic parameters of ammonium, nitrate, and phosphate uptake curves in the three species considered in this study.

**Nutrient**		*****S. perennis*** ssp. ***alpini*****	***A. portulacoides***	***A. macrostachyum***
${\text{NH}}_{4}^{+}$	V_max_ (μmol g^−1^ DWmin^−1^)	3.31 (0.27)^a^	6.95 (0.49)^b^	1.74 (0.40)^c^
	K_m_ (μM)	1,164.5 (163.38)^a^	3,129.9 (126.7)^b^	378.65 (133.81)^c^
	CP (μM)	34.49 (6.15)^a^	80.86 (11.16)^b^	78.65 (13.81)^b^
${\text{NO}}_{3}^{-}$	V_max_ (μmol g^−1^DWmin^−1^)	0.24 (0.01)^a^	0.25 (0.06)^a^	0.30 (0.05)^a^
	K_m_ (μM)	19.75 (3.18)^a^	45.43 (4.68)^b^	15.67 (6.13)^a^
	CP (μM)	2.33 (0.39)^a^	0.25 (0.10)^b^	3.17 (0.88)^a^
${\text{PO}}_{4}^{3-}$	V_max_ (μmol g^−1^DWmin^−1^)	0.76 (0.17)^a^	0.30 (0.03)^b^	1.83 (0.04)^c^
	K_m_ (μM)	158.89 (21.49)^a^	32.15 (10.60)^b^	247.89 (20.77)^c^
	CP (μM)	6.72 (1.63)^a^	1.47 (0.58)^b^	8.78 (1.38)^a^

*Standard deviations are shown in parentheses (n = 4). For each parameter, values of the species with the same letter in the superscript are not significantly different (p > 0.05)*.

We also compared the nutrient uptake rates of the three species at the most frequent concentrations found in the salt marsh, pointed out by arrows in Figure 5. At the low ammonium concentrations mostly observed in the salt marsh (100–400 μM, a thick arrow, Figure 5A), *S. perennis* ssp. *alpini* presented an average uptake rate of 0.43 ± 0.07 μmoles g DW min^−1^, almost 2-fold higher than the other two species, whereas, at the high submodal concentration (around 900 μM, a thin arrow in Figure 5A), *S. perennis* ssp. *alpini* and *A. portulacoides* showed the highest uptake rate, (1.47 ± 0.19 and 1.56 ± 0.17 μmoles g DW min^−1^, respectively). Nitrate uptake rate at the modal concentration around 2.5 μM was very low in *A. portulacoides* (0.008 μmols g DW min^−1^), but notably higher than in *S. perennis* ssp. *alpini* and *A. macrostachyum*, which were practically null (Figure 5B). At the most common concentrations of phosphate in the salt marsh (10–20 μM, an arrow in Figure 5C), *S. perennis* ssp. *alpini* and *A. portulacoides* displayed higher uptake rates of 0.08 and 0.1 μmols g DW min^−1^, respectively, while, in *A. macrostachyum*, was only 0.02 μmoles g DW min^−1^.

### Effect of Salinity on Nutrient Uptake

**Figure 6:**
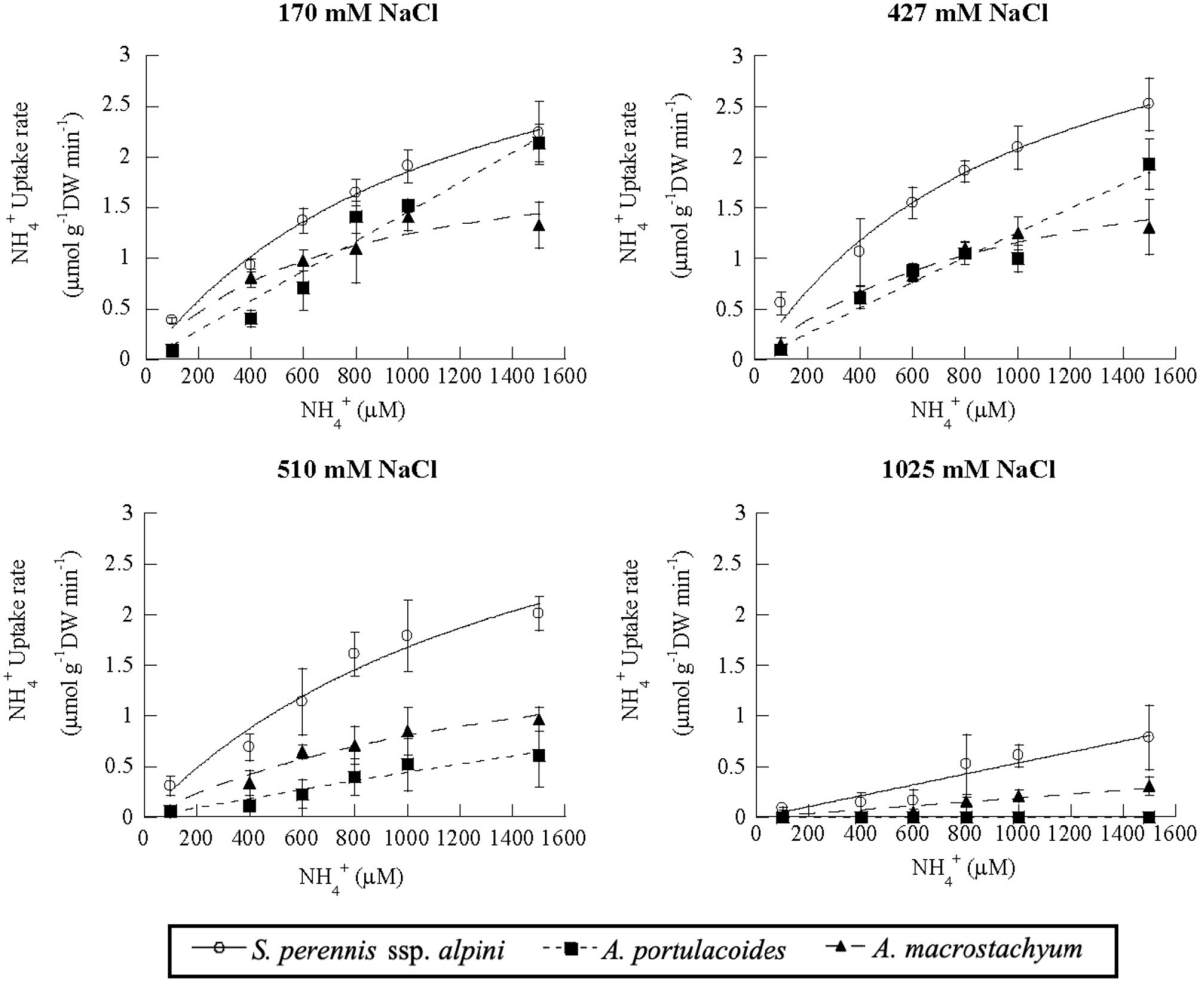
Uptake kinetics of ammonium in *S. perennis* ssp. *alpini, A. portulacoides* and *A. macrostachyum* at different salinities (170, 427, 510, and 1,025 mM NaCl). Bars represent standard deviations (*n* = 4).

**Figure 7:**
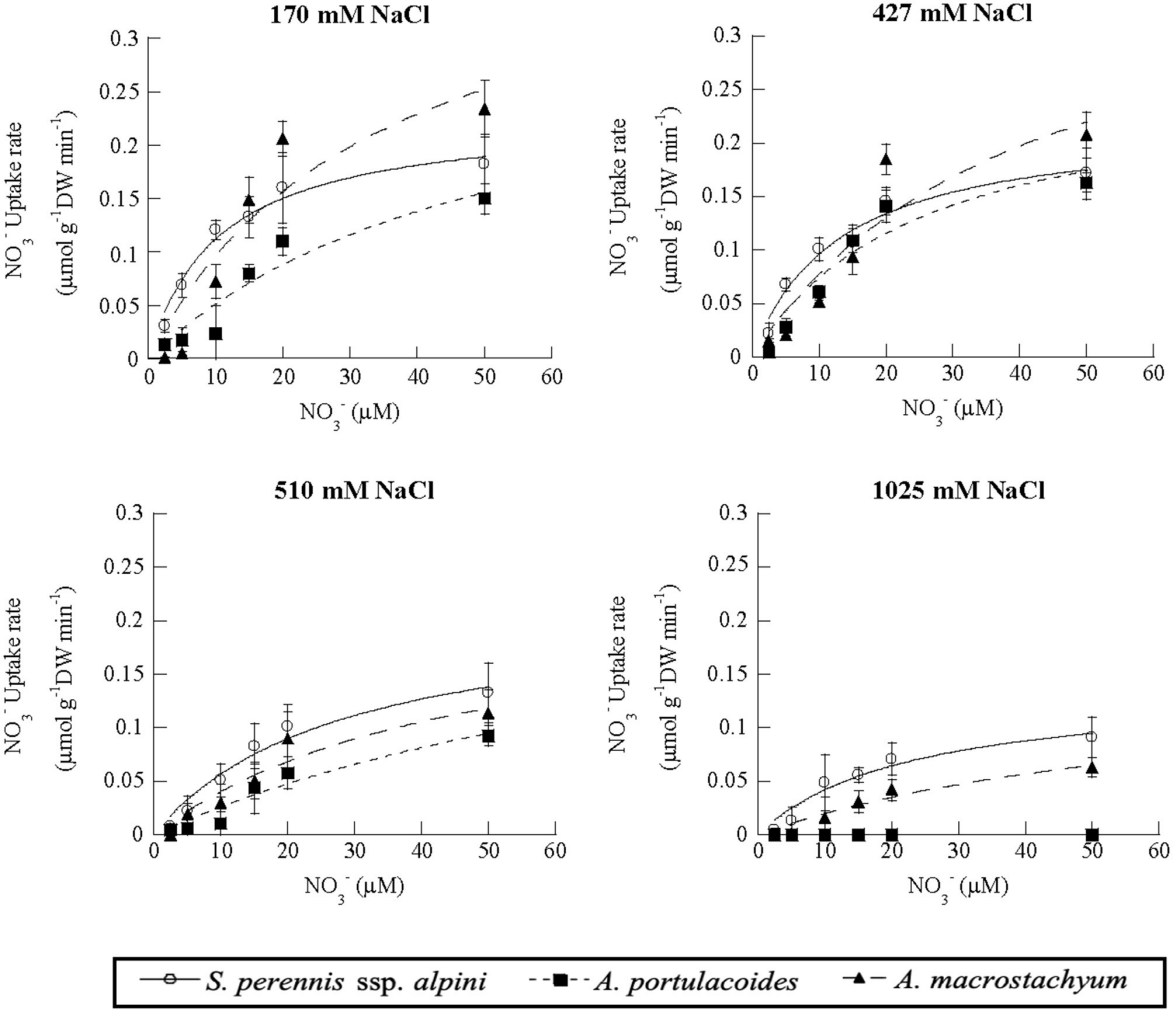
Uptake kinetics of nitrate in *S. perennis* ssp. *alpini, A. portulacoides* and *A. macrostachyum* at different salinities (170, 427, 510, and 1,025 mM NaCl). Bars represent standard deviations (*n* = 4).

**Figure 8:**
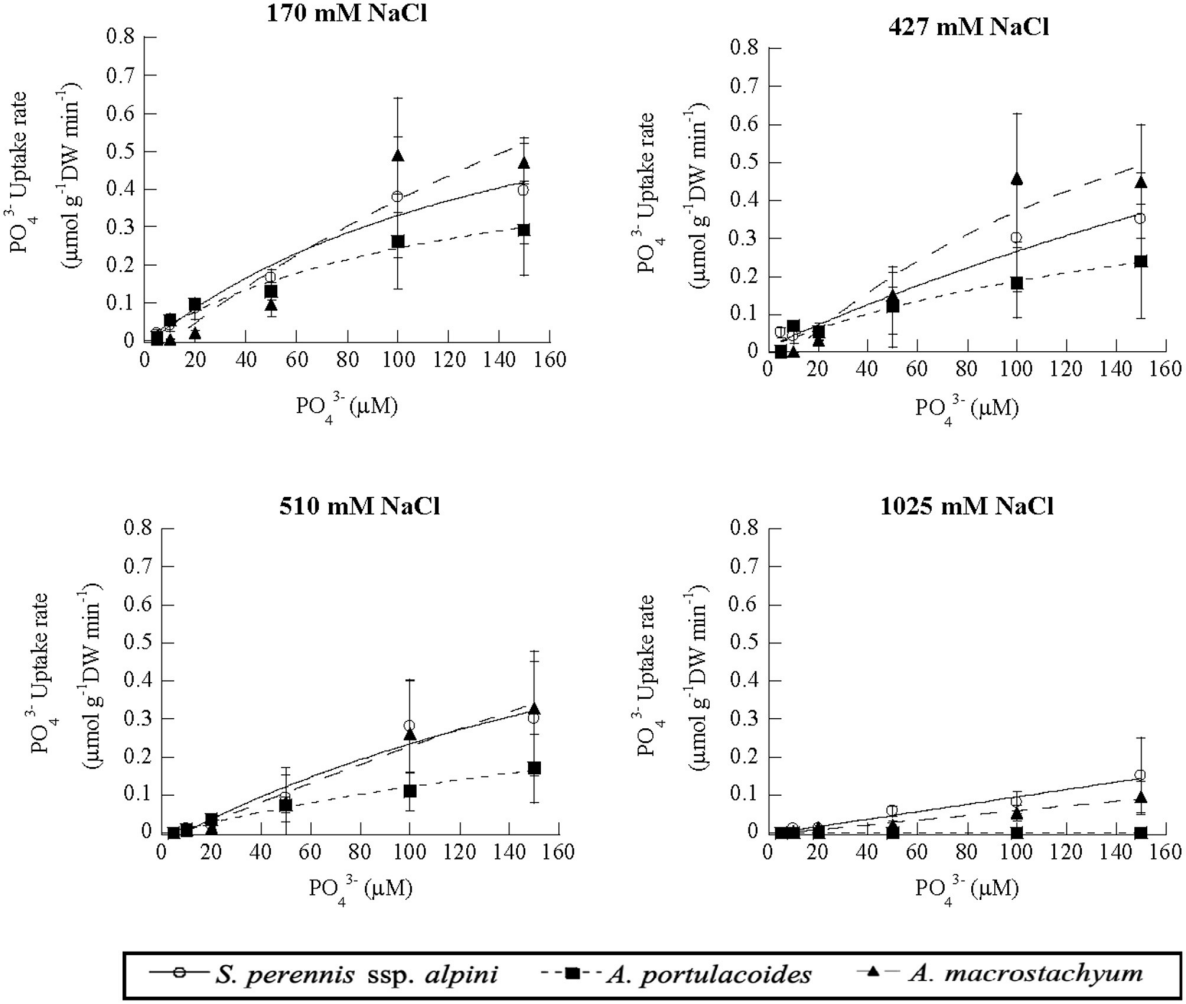
Uptake kinetics of phosphate in *S. perennis* ssp. *alpini, A. portulacoides* and *A. macrostachyum* at different salinities (170, 427, 510, and 1,025 mM NaCl). Bars represent standard deviations (*n* = 4).

Salinity influenced nutrient uptake differently in the three studied species of the salt marsh (Figures 6–**8**). Maximum uptake rates (V_max_) of ammonium were similar at all salinities except for the highest one (1,025 mM NaCl) in *S. perennis* ssp. *alpini* and *A. macrostachyum* (Table 2). At this high salinity, uptake kinetics in both species showed a linear response (Figure 6). The maximum uptake rate, observed at 1,500 μM ${\text{NH}}_{4}^{+}$, was reduced by 82% in *S. perennis* ssp. *alpini* and 83% in *A. macrostachyum* (Table 2). On the other hand, CP increased in *S. perennis* ssp. *alpini* at 427 mM NaCl, as well as K_m_ in *A. macrostachyum* at 510 mM NaCl. The most affected species by increasing salinity was *A. portulacoides*, since its V_max_ for ammonium notably decreased already at 427 mM NaCl, although K_m_ was not affected by salinity. What is more, this species was unable to take up any nutrient and withered at the highest salinity assayed (Table 2).

**Table 2:** Kinetic parameters of ammonium uptake curves measured at different salinities in the three species of this study.

**Salinity** **mM NaCl**		*****S. perennis*** ssp. ***alpini*****	***A. portulacoides***	***A. macrostachyum***
170	V_max_ (μmol g^−1^DWmin^−1^)	4.36 (0.68)^a^	5.74 (0.81)^a^	1.77 (0.25)^a^
	K_m_ (μM)	1,417.9 (457.45)^a^	1,798.7 (178.8)^a^	305.6 (186.45)^a^
	CP (μM)	25.54 (13.41)^a^	92.94 (61.59)^a^	80.01 (27.96)^a^
427	V_max_ (μmol g^−1^DWmin^−1^)	5.31 (1.06)^a^	4.54 (0.31)^b^	2.04 (0.33)^a^
	K_m_ (μM)	1,805.2 (687.6)^a^	1,675.1 (321.3)^a^	671.33 (302.1)^a^
	CP (μM)	87.81 (53.39)^b^	83.5 (11.31)^a^	48.52 (31.25)^a^
510	V_max_ (μmol g^−1^DWmin^−1^)	4.33 (1.23)^a^	3.61 (0.53)^b^	2.03 (0.57)^a^
	K_m_ (μM)	1,499.1 (730.2)^a^	1,228.6 (329.1)^a^	1,530.8 (708.1)^b^
	CP (μM)	81.72 (12.36)^b^	60.66 (13.95)^a^	74.38 (42.05)^a^
1,025	V_1500μM_ (μmol g^−1^DWmin^−1^)	0.79 (0.32)^b^	–	0.31 (0.09)^b^

*Standard deviations are shown in parentheses (n = 4). The symbol – indicates that A. portulacoides withered at that salinity. Statistical comparisons of the parameters are made among salinities for each species, and values with the same letter in the superscript are not significantly different (p > 0.05)*.

Nitrate uptake showed saturation kinetics within the concentration range assayed at all salinities (Figure 7). At 1,025 mM NaCl, maximum uptake rates decreased by 41 and 81% in *S. perennis* ssp. *alpini* and *A. macrostachyum*, respectively, and *A. portulacoides* withered, as occurred in the ammonium treatment (Table 3). The other kinetic parameters were not affected by salinity, except for K_m_ of *S. perennis* ssp. *alpini* and *A. macrostachyum*, which increased at 510 mM NaCl.

**Table 3:** Kinetic parameters of nitrate uptake curves measured at different salinities in the three species of this study.

**Salinity** **mM NaCl**		*****S. perennis*** ssp. *alpini***	***A. portulacoides***	***A. macrostachyum***
170	V_max_ (μmol g^−1^DWmin^−1^)	0.22 (0.02)^a^	0.31 (0.14)^a^	0.42 (0.18)^a^
	K_m_ (μM)	10.47(2.17)^a^	51.71 (16.49)^a^	34.27 (26.12)^a^
	CP (μM)	3.45 (1.22)^a^	2.02 (0.34)^a^	3.36 (1.13)^a^
470	V_max_ (μmol g^−1^DWmin^−1^)	0.22 (0.02)^a^	0.26 (0.06)^a^	0.40 (0.10)^a^
	K_m_ (μM)	12.97 (3.31)^a^	25.22 (13.08)^a^	41.96 (13.67)^a^
	CP (μM)	1.16 (0.86)^a^	2.02 (1.23)^a^	2.46 (2.24)^a^
510	V_max_ (μmol g^−1^DWmin^−1^)	0.21 (0.03)^a^	0.16 (0.05)^a^	0.22 (0.08)^ab^
	K_m_ (μM)	27.90 (9.73)^b^	70.38 (19.14)^b^	45.46 (29.73)^a^
	CP(μM)	2.13 (0.65)^a^	2.33 (0.25)^a^	2.60 (1.54)^a^
1,025	V_max_ (μmol g^−1^DWmin^−1^)	0.13 (0.02)^b^	–	0.08 (0.03)^b^
	K_m_ (μM)	10.34 (4.25)^a^	–	64.79 (44.90)^a^
	CP (μM)	2.32 (0.62)^a^	–	5.31 (2.61)^a^

*Standard deviations are shown in parentheses (n = 4). The symbol – indicates that A. portulacoides withered at that salinity. Statistical comparisons of the parameters are made among salinities for each species, and values with the same letter in the superscript are not significantly different (p > 0.05)*.

Uptake kinetics of phosphate showed saturation at lower salinities, but were linear at 1,025 mM Na Cl in *S. perennis* ssp. *alpini* and *A. macrostachyum* (Figure 8). At this high salinity, V_max_, observed at 150 μM ${\text{PO}}_{4}^{3-}$, was reduced by 85% in *S. perennis* ssp. *alpini* and 95% in *A. macrostachyum* (Table 4). The other kinetic parameters of phosphate uptake were not affected by salinity within the range of concentrations assayed in both species. As it occurred for nitrogenous nutrients, the highest salinity treatment prevented *A. portulacoides* from taking up phosphate from the medium and its affinity for this nutrient decreased already at 427 mM Na Cl.

**Table 4:** Kinetic parameters of phosphate uptake curves measured at different salinities in the three species of this study.

**Salinity** **mM NaCl**		*****S. perennis*** ssp. ***alpini*****	***A. portulacoides***	***A. macrostachyum***
170	V_max_ (μmol g^−1^ DWmin^−1^)	0.99 (0.4)^a^	0.28 (0.12)^a^	1.89 (0.20)^a^
	K_m_ (μM)	203.69(127.54)^a^	23.01 (13.04)^a^	183.2 (88.8)^a^
	CP (μM)	2.68 (0.64)^a^	2.92 (2.30)^a^	10.79 (1.31)^a^
427	V_max_ (μmol g^−1^ DWmin^−1^)	1.29 (0.55)^a^	0.26 (0.18)^a^	1.67 (0.51)^a^
	K_m_ (μM)	156.5 (89.7)^a^	98.33 (39.33)^b^	261.5 (89.7)^a^
	CP (μM)	5.23 (1.25)^a^	16.88 (3.43)^b^	4.08 (1.01)^a^
510	V_max_ (μmol g^−1^ DWmin^−1^)	0.99 (0.36)^a^	0.19 (0.05)^a^	1.60 (0.26)^a^
	K_m_ (μM)	286.2 (123.4)^a^	91.36 (20.77)^b^	351.2 (91.2)^a^
	CP (μM)	8.93 (6.59)^a^	5.07 (1.29)^a^	9.99 (5.88)^a^
1,025	V_150μM_ (μmol g^−1^DWmin^−1^)	0.15 (0.10)^b^	–	0.095 (0.04)^b^

*Standard deviations are shown in parentheses (n = 4). The symbol - indicates that A. portulacoides withered at that salinity. Statistical comparisons of the parameters are made among salinities for each species, and values with the same letter in the superscript are not significantly different (p > 0.05)*.

## Discussion

Our results reveal that Chenopodiacean plants studied have different uptake kinetic performances for ammonium, nitrate, and phosphate in hydroponic cultures, and that the effect of increasing salinity on them is species-specific. Kinetic parameters and nutrient uptake rates at real concentrations observed in the field may partly explain the local dominance of plant species. Salinity appears to be a stressor that, at high values, negatively affects nutrient uptake and thus could determine the growth and survival of the saltmarsh species. This approach of using short-term nutrient uptake measurements as a physiological variable to explain plant distribution and abundance in salt marshes has scarcely been used (Mozdzer et al., 2010; MacTavish and Cohen, 2017; Cott et al., 2018). However, the importance of knowing the changes in the kinetics of nutrient uptake and differential species responses has been pointed out as critical to predicting ecosystem responses to global change (Bassirirad, 2000).

High nutrient concentrations found in the sediment interstitial water of Palmones salt marsh are in agreement with the eutrophication pattern observed from the last three decades. Since the early 1990s, nitrogen and phosphorus loads have increased in the estuary and consequently in the associated salt marsh (Clavero et al., 1999, 2000; Rubio et al., 2003; Palomo and Niell, 2009), mainly due to the lower river flow, coupled with the enhancement of nutrient enrichment by tidal fertilization (Clavero et al., 1999, 2000). Eutrophic processes in salt marshes have been associated with the increase of ${\text{NO}}_{3}^{-}$ in the water coming from human activities (Deegan et al., 2007, 2012); however, ammonium is the key nitrogen species in this study, as nitrate concentrations have progressively decreased since 2000 down to most frequent values lower than 2 μM. This fact can be related to the reduction in agriculture areas close to the salt marsh, together with the abandonment of nitrate as a fertilizer and the interruption of denitrification processes in the sediment (Niell et al., 2005; Arrojo, 2012). On the contrary, ammonium concentration has kept increasing, also due to urban waste waters nearby and the accumulation of the organic matter in the sediment coming from the enhanced primary production in the salt marsh (Palomo and Niell, 2009).

To our knowledge, this is the first description of nutrient uptake kinetics in the three halophytic species, besides the work of two of the authors, carried out with excised roots of *S. perennis* (Muñoz and Niell, 2009). We attempted to use nutrient uptake kinetics from our study to understand plant distribution and competition among the studied species. We acknowledge that uptake physiology is affected by the physical and chemical microenvironment in the rhizosphere (Mendelssohn and Morris, 2000); however, our incubation was done under the same culture conditions, allowing for comparisons among species. Uptake rates of nitrogen and phosphate at concentrations commonly found in the marsh were much lower than the V_max_ from the Michaelis-Menten model for all species, since their modal values of substrate concentration in the salt marsh were in the lower ranges. Maximal uptake capacity at saturation (V_max_) for ammonium was obtained in *A. portulacoides*, 2- and 4-fold higher than in *S. perennis* ssp. *alpini* and *A. macrostachyum*, respectively. However, the theoretical concentration for reaching that V_max_ is never observed in the salt marsh. In fact, at the most frequently observed concentrations (100–400 μM), the species with the highest uptake rate was *S. perennis* ssp. *alpini*. These results suggest that *S. perennis* ssp. *alpini* is favored at the current most probably found ammonium concentrations, while *A. portulacoides* would outcompete the other two species at higher nutrient levels. That high V_max_ of *A. portulacoides* indicates that it is a high-nutrient species, and thus it could take advantage under N enrichment conditions (Cott et al., 2018). In this regard, Álvarez-Rogel et al. (2007) considered the possibility of the observed expansion of that species was influenced by external inputs of eutrophicated waters in another Mediterranean salt marsh. Values of maximum uptake capacity and affinity in *S. perennis* ssp. *alpini* were lower than those reported by Muñoz and Niell (2009). This discrepancy can be explained because we used intact plants, instead of excised roots, which can overestimate uptake rates (Falkengren-Grerup et al., 2000). On the other hand*, A. macrostachyum* with low V_max_ and an uptake rate at frequent ammonium concentrations and higher affinity (lower K_m_) appears to be less competitive in relation to N uptake in the outer zone, where *S. perennis* ssp. *alpini* and *A. portulacoides* predominate. Maximum capacity for nitrate uptake can be discarded as a differential parameter, as all species presented similar V_max_ values, but *S. perennis* ssp. *alpini* and *A. macrostachyum* showed a higher affinity for this nutrient. Nevertheless, ammonium can be considered the main N source used by the studied species, as uptake rates were from 6- up to 28-fold greater than for nitrate, a pattern also observed in other saltmarsh plants (Mozdzer et al., 2011; Cott et al., 2018). This noticeable difference can be attributed to the much higher ammonium concentration available for the plants (almost two orders of magnitude) and the relatively high energetic cost of reducing and assimilating ${\text{NO}}_{3}^{-}$ compared with ammonium (Lambers et al., 1998). The species with the highest V_max_ for phosphate was *A. macrostachyum*, although the most frequent phosphate concentrations in the salt marsh never reach the theoretical ones for that V_max_. In fact, *S. perennis* ssp. *alpini* and *A. portulacoides* showed greater uptake rates than *A. macrostachyum* at modal 10–20 μM phosphate. In the inner zone, higher concentrations can also be observed (submodal values of 50–70 μM phosphate), where *A. macrostachyum* is more abundant and could be favored for phosphate uptake. These results suggest that *S. perennis* ssp. *alpini* and *A. portulacoides* are better competitors at the current lower phosphate concentrations, but *A. macrostachyum* could expand when the phosphate load increased. Because of these specific differences in nutrient uptake performance, we expect that increasing coastal eutrophication might modify marsh plant community structure by altering species competitive balance (Levine et al., 1998; Bertness and Ewanchuk, 2002; Pennings et al., 2005). Nevertheless, other environmental factors and the interactions cannot be discarded in explaining the distribution and composition of the saltmarsh plant community (Pennings and Callaway, 1992; Fariña et al., 2009).

Halophytes are characterized by their ability to thrive in saline environments above 200-mM NaCl (Flowers et al., 1986; Flowers and Colmer, 2008). In our study, salinities higher than 170 and 517 mM NaCl, depending on the species, appear as a stressing factor and caused a loss of nutrient uptake capacity. This is consistent with studies that observed a decrease in nutrient uptake rates in saltmarsh plants under high saline conditions (Morris, 1984; Mozdzer et al., 2010; MacTavish and Cohen, 2017), although a positive effect at moderate levels has also been reported (Bradley and Morris, 1991). In Palmones salt marsh, *A. portulacoides* was the most sensitive species, decreasing its V_max_ for ammonium, the main nitrogen source, in an average value of 40% at already 427 mM NaCl and not tolerating the highest salinity of 1,025 mM NaCl, where it withered. This result agrees with the drastic reduction in growth rates by salinities of 410–690 mM NaCl (Jensen, 1985) and, also, photosynthetic rates by 400–700 mM NaCl (Redondo-Gómez et al., 2007b). In this genus, a wide range of salinity values has been reported for a maximum growth rate, such as 85–200 mM NaCl in *A. atriplex* (Jensen, 1985; Redondo-Gómez et al., 2007b), 340–427 mM NaCl in *A. centralasiatica* (Qui et al., 2003), 340–850 mM NaCl in *A. amnicola* (Aslam et al., 1986), 600 mM NaCl in *A. inflata* and *A. nummulari* (Ashby and Beadle, 2000), evidencing high plasticity of the genus in response to local conditions. Thus, *A. portulacoides* would be the least resistant species compared with others in the genus in agreement with the observed physiological response of our study. In contrast, *S. perennis* ssp. *alpini* and *A. macrostachyum* cope better with increasing salinity, showed by the maintained nutrient uptake rates up to 510 mM NaCl. Our results support the well described adaptation of *A. macrostachyum* to extremely high-salinity soils. In fact, its distribution in the inner zone of the salt marsh has been extensively related to the resistance to high salinity (Curcó et al., 2002; Redondo-Gómez et al., 2010; González-Alcaraz et al., 2014; Vélez-Martín et al., 2020).

This species has a broad optimum of 171–510 mM NaCl for growth and net photosynthesis (Redondo-Gómez et al., 2010), consistently with the greater biomass production of 200–400 mM NaCl reported by Khan et al. (2005). Even more, the low nutrient uptake rates of *A. macrostachyum* at 1,025 mM NaCl from our study are in agreement with the drastic photosynthesis decrease observed by Redondo-Gómez et al. (2010). *Sarcocornia perennis* ssp. *alpini* also presented extreme tolerance to salinity, with high uptake rates up to 510 mM NaCl, similarly to *S. perennis* (Adams and Bate, 1994). A comparable growth response to high salinity was seen in *Sarcocornia fruticosa* (L) A.J. Scott, another chenopod abundant in salt marshes of SW Spain (Redondo-Gómez et al., 2006). On the other hand, the higher nitrogen uptake rates and slightly greater salinity resistance at 1,025 mM NaCl in *S. perennis* ssp. *alpini* than in *A. macrostachyum* could be a competitive advantage for the former species in a future scenario of increasing salinization. At present, the dominance of *A. macrostachyum* in the inner zone is probably related to its low tolerance to waterlogging and elevation preference, which seem to be critical to its survival in Mediterranean wetlands (Ibañez et al., 1999; Curcó et al., 2002; Redondo-Gómez et al., 2010; Vélez-Martín et al., 2020).

The negative effect of salinity on plant growth has been related to water stress, ion toxicities, ion imbalance, or a combination of these factors (Waisel, 1972; Adam, 1990; Ungar, 1991). In *A. portulacoides* and *A. macrostachyum*, reduction in photosynthesis at 700 and 1,030 mM NaCl, respectively, was accounted for largely by limitation by stomal and mesophyll conductance and intercellular CO_2_ (Redondo-Gómez et al., 2007b, 2010). Moreover, at a cellular scale, the lower plasma membrane Na^+^ permeability in *S. perennis* spp. *alpini* in comparison to *A. portulacoides* (Rubio and Fernández, 2019) might be also related to the more resistance to extreme salinity in the former species, since the uptake of Na^+^ depolarizes the membrane, especially under excess concentrations, preventing the entrance of other ions, such as ammonium.

In the present study, we analyzed nutrient uptake physiology to understand the abundance of the three studied species in Palmones salt marsh. Nutrient uptake can be considered a good estimate for growth response. In this regard, Cott et al. (2018), based on the comparison of ^15^N uptake rates in hydroponic cultures and long-term field biomass data, suggested that N uptake kinetics may underlie the strong productivity response of plants to N in the salt marsh. Likewise, Veldhuis et al. (2019) found a good correlation between growth rates in laboratory experiments and the abundance of plants in the field. Estimations of biomass values for the three species studied in Palmones salt marsh were done by Palomo (2004) and Palomo and Niell (2009). *Sarcocornia perennis* ssp. *alpini* showed the greatest values, with aboveground biomass averaging 3,420 g DW m^−2^ (Palomo and Niell, 2009), followed by *Atriplex portulacoides*, with average biomass of 2,270 g DW m^−2^, whereas *A. macrostachyum* was the least-abundant species, with average biomass of 1,400 g DW m^−2^ (Palomo, 2004). Therefore, the high uptake rates at ammonium and phosphate modal concentrations of *Sarcocornia perennis* ssp. *alpini* obtained in our experiments could support its greater abundance in the salt marsh, especially in relation to *A. macrostachyum*, which showed the lowest rates. It is also noteworthy that species biomass was higher than in other European populations (Cartaxana and Catarino, 1997; Ibañez et al., 1999, 2004; Bouchard and Lefeuvre, 2000; Figueroa et al., 2003; Crain, 2007), which might be related to eutrophication increase in the Palmones salt marsh (Niell et al., 2005; Palomo and Niell, 2009) and the high water renewal rate of the estuary (Sánchez de Pedro et al., 2013).

Experimental studies have demonstrated the role of competition in the plant distribution pattern along the gradients in macrotidal salt marshes, particularly in low stressful environments (Grime, 1979; Bertness, 1991; Pennings and Callaway, 1992; Craine, 2005). On the other hand, nutrient supply and resource competition have been shown to interact with physical stress in salt marshes, especially under conditions of nitrogen limitation (Levine et al., 1998; Emery et al., 2001). Despite ammonium and phosphate in Palmones salt marsh soil are in excess, competition may exist through differential responses in nutrient uptake and subsequent growth. The uptake kinetics obtained in this study and high biomass values (Palomo, 2004; Palomo and Niell, 2009) suggest that current nutrient concentrations do not represent a stress factor for the three chenopods, and that there is displacement in nutrient exploitation capacity. In this sense, *S. perennis* ssp. *alpini* has advantage at lower, most frequently found ammonium concentrations, whereas *A. portulacoides* performs better at higher ones, both cohabiting the outer zone and outcompeting *A. macrostachyum* that showed the lowest uptake capacity. On the other hand, the latter species is favored at higher phosphate concentrations, more usually found in the inner zone, while *S. perennis* ssp. *alpini* and *A. portulacoides* have greater uptake capacity at lower values. One explanation for the segregation of *A. macrostachyum* toward the inner zone could be that this species is displaced to the more physically stressful habitat by the other two competitive dominant species as a trade-off between stress tolerance and competitive ability, observed in other saltmarsh plant communities (Bertness, 1992; Pennings and Bertness, 2001; Pennings et al., 2005). However, the zonation in Palmones cannot entirely be explained by that hypothesis, probably because of the lack of a clear gradient in physical stress across the marsh (Costa et al., 2003), at least in terms of salinity. In this regard, Batriu et al. (2011) suggested that facilitation and competition would play a more important role than environmental gradients in zonation in Mediterranean coastal marshes.

The choice between resilience and transformation or loss of salt marshes as ecosystems depends on their adaptive response to a series of disturbances that change anachronistically over space and time (Staudt et al., 2013). In this context, understanding the effect of global change factors, such as eutrophication and salinity, on salt marsh primary producers is needed for managing these valuable ecosystems. Overall, our results suggest that in a future scenario of progressive enhancement of an ammonium load in the saltmarsh sediment, *A. portulacoides* would be more competitive for nitrogen and could expand in the outer zone. In fact, this seems to have started to occur in the last years (pers. obs.). However, the increasing salinization predictions of coastal wetlands would hamper this expansion by decreasing nutrient uptake. Likewise, an increase in phosphorus concentration would favor the growth of *A. macrostachyum* in the inner zone. On the other hand, nutrient uptake and salinity, albeit being factors of paramount importance in controlling primary production, cannot entirely explain the distribution of halophytes in Palmones salt marsh. Therefore, other variables, such as waterlogging, anoxia, and local elevation (Bennet et al., 2009; Mossman et al., 2020; Vélez-Martín et al., 2020), must be considered in order to complement our observations. Moreover, further research is needed to study if growth and photosynthetic capacity of the studied species actually reflect the differential response of nutrient uptake reported here. The study of the combination of those multiple factors (Silvestri et al., 2005) will help us to understand the functioning of this saltmarsh plant community and predict possible changes in response to factors related to global change.

## Data Availability Statement

The raw data supporting the conclusions of this article will be made available by the authors, without undue reservation.

## Author Contributions

FN conceived and designed the research project, supervised the work, and participated in writing the manuscript. RM planned and performed field and laboratory work. RM and RC analyzed the data. RC wrote the manuscript. All authors approved the final version of the manuscript.

## Conflict of Interest

The authors declare that the research was conducted in the absence of any commercial or financial relationships that could be construed as a potential conflict of interest.

## Publisher's Note

All claims expressed in this article are solely those of the authors and do not necessarily represent those of their affiliated organizations, or those of the publisher, the editors and the reviewers. Any product that may be evaluated in this article, or claim that may be made by its manufacturer, is not guaranteed or endorsed by the publisher.
